# Effectiveness of High-Intensity Electromagnetic Stimulation Delivered by the Super Inductive System Combined with Therapeutic Exercise and Health Education in Lateral Epicondylalgia: Study Protocol for a Multicenter Randomized Controlled Trial

**DOI:** 10.3390/life16101596

**Published:** 2026-09-24

**Authors:** María del Carmen Sánchez-Mato, Ramon Arroyo-Aljaro, Jordi Alcaide-Altet, Ana M. Gómez-Gil, Luisa Acosta-Moreno, Sara Izquierdo-Cuesta, Rosa Maria Garcia-Sierra, Anna Escribà-Salvans

**Affiliations:** 1 Primary Care Rehabilitation Center, Institut Català de la Salut (ICS), 08303 Mataró, Spain; mariadelcarmen.sanchez@uvic.cat (M.d.C.S.-M.); jordi.alcaide@uvic.cat (J.A.-A.); amgomez.ics@gencat.cat (A.M.G.-G.); 2 Research Group on Methodology, Methods, Models and Results of the Social and Health Sciences (M3O), Faculty of Health and Welfare Sciences, University of Vic-Central University of Catalonia (UVic-UCC), 08500 Vic, Spain; 3 Primary Care Rehabilitation Center, Institut Català de la Salut (ICS), 08030 Barcelona, Spain; rarroyo.bcn.ics@gencat.cat (R.A.-A.); lacosta.bcn.ics@gencat.cat (L.A.-M.); saraizquierdo.bcn.ics@gencat.cat (S.I.-C.); 4 Research Support Unit Metropolitana Nord, Primary Care Research Institut Jordi Gol (IDIAPJGol), 08303 Mataró, Spain; rgarciasi.mn.ics@gencat.cat; 5 Multidisciplinary Research Group in Health and Society GREMSAS (2021 SGR 01484), 08007 Barcelona, Spain; 6 Institute for Research and Innovation in Life and Health Sciences of Central Catalonia (IRIS-CC), 08500 Vic, Spain

**Keywords:** lateral epicondylalgia, Super Inductive System, high-intensity electromagnetic stimulation, exercises, pain, functionality

## Abstract

Background: Lateral epicondylalgia is a common musculoskeletal condition that negatively affects functionality and quality of life, with a clinical and socioeconomic impact. Although therapeutic exercise is considered the first-line conservative treatment, there is no consensus on the most effective strategy, and the role of electrotherapy techniques remains controversial. Objectives: The objective of this study is to evaluate the effectiveness of the Super Inductive System (SIS) combined with a therapeutic exercise and health education program in patients with lateral epicondylalgia. Methods: A randomized, multicenter, blinded trial is proposed. A total of 120 participants will be randomly assigned to an intervention group (IG) or a control group (CG). Participants in the IG will receive SIS treatment at a therapeutic dose, combined with therapeutic exercise and health education. Participants in the CG will receive SIS at a non-therapeutic dose along with the same exercise and education program. The intervention will consist of eight sessions. The primary outcome will be the PRTEE score. Grip strength, mobility, and quality of life will be assessed at baseline, immediately after treatment, and at 3- and 6-month follow-up assessments. This study will evaluate the effectiveness of SIS in lateral epicondylalgia.

## 1. Introduction

Tendinous diseases constitute a significant portion of musculoskeletal disorders and are a major cause of disability worldwide. Unresolved chronic inflammatory processes have been associated with the onset and progression of tendinous pathologies, promoting undesirable immune responses and structural alterations of the tissues [1].

Lateral epicondylalgia (LE) is a common musculoskeletal disorder characterized by pain and dysfunction in the upper extremity, and is considered the most common cause of elbow pain [2,3]. Although the term “epicondylitis” is widely used, this condition is not truly inflammatory but degenerative, resulting from a tendinopathy process associated with overuse and repetitive loads [4].

This condition primarily affects the wrist extensor muscles, especially the short head of the extensor carpi radialis brevis muscle, although other extensor muscles may also be involved. It occurs in both athletes and individuals who perform repetitive work activities involving wrist extension, supination, and grasping, leading to limitations in activities of daily living (ADL) and work performance [2,3].

LE is a significant public health issue, closely associated with occupational factors such as demanding physical work and repetitive movements [5,6]. It affects approximately 1% to 3% of the population, with an incidence of about 5.5 cases per 1000 people per year, predominating in individuals aged 40 to 59 and usually affecting the dominant limb [3,7]. Symptomatology includes localized pain, decreased strength, functional impairment, and a negative impact on quality of life [8].

The diagnosis is primarily clinical and is based on the presence of pain over the lateral epicondyle reproduced by provocative clinical tests such as the Cozen test and the chair test [9]. In some cases, it may be supplemented with imaging tests such as ultrasound [9,10]. Pain is assessed using validated scales such as the Visual Analog Scale (VAS) or the numerical rating scale (NRS), while grip strength can be measured with dynamometry [3,10,11].

Despite the wide range of available treatment options, there is currently no consensus on the most effective therapeutic strategy for LE [12]. Rest and pharmacological treatment can relieve pain in the short term, although they can also contribute to relapses [13].

Therapeutic exercise, especially eccentric training, has been established as a fundamental intervention in the treatment of epicondylalgia, demonstrating improvements in pain, strength, and functionality [3,13,14,15]. Eccentric exercise is considered one of the main conservative treatment strategies, with a favorable cost–benefit ratio. In this context, the progressive increase in tendinous load through isometric, strength, and velocity exercises has been proposed as an effective strategy to optimize recovery [16,17,18].

Various physiotherapy modalities have been used in the treatment of LE. Electrotherapy and thermotherapy have shown beneficial effects in reducing pain and improving function, especially when combined with therapeutic exercise and manual techniques [3,7]. Different modalities such as Transcutaneous Electrical Nerve Stimulation (TENS), laser, electromagnetic fields, and ultrasound therapy are widely used in clinical practice. A systematic review and meta-analysis reported significant clinical improvements with the combination of physical therapy and electrotherapy compared with placebo [18]. Similarly, pulsed electromagnetic field (PEMF) therapy has been associated with reduced pain and improved grip strength [12]. However, many of these reported benefits have been observed primarily in the short term, and evidence regarding their medium- and long-term effectiveness remains limited. Furthermore, most published evidence refers to conventional low-intensity PEMF devices, whose technical characteristics and physiological mechanisms differ substantially from those of the Super Inductive System (SIS). Therefore, studies including longer follow-up periods are needed to determine whether treatment effects are sustained over time [12].

The SIS, based on the application of high-intensity electromagnetic stimulation, has emerged as an innovative therapeutic technology [19]. Unlike conventional low-intensity PEMF devices, SIS is capable of inducing depolarization of peripheral nerves and visible muscle contractions, which may reflect different physiological mechanisms and clinical effects. Some preliminary studies suggest an immediate reduction in pain following its application, with effects that may persist in the short term. Although pulsed electromagnetic fields have shown potential in modulating inflammatory processes and tissue regeneration, as well as in improving tendon structure and muscle recovery [1,20], these findings should be interpreted with caution when extrapolated to SIS interventions because of the differences in stimulation intensity and underlying physiological mechanisms.

Other interventions such as manual therapy, exercise programs, kinesiotaping, and the use of orthoses, extracorporeal shock waves, or dry needling have also been used with variable results [3,21,22,23,24,25,26,27,28,29]. However, the effectiveness of these therapies remains a matter of debate, with heterogeneous and, in some cases, limited evidence.

Given the lack of consensus on conservative treatment and the limited evidence regarding the use of SIS, it is necessary to develop studies with rigorous methodological designs that allow for the evaluation of its therapeutic potential within a multimodal approach. In this context, the aim of the present study is to evaluate the effectiveness of the SIS combined with a therapeutic exercise and health education program compared with an SIS intervention at a non-therapeutic dose in patients with LE [19,30].

## 2. Materials and Methods

### 2.1. Study Design and Setting

This study protocol was prospectively registered at ClinicalTrials.gov (NCT07495254) on 22 March 2026, prior to the enrolment of the first participant. Participant recruitment commenced on 30 March 2026.

An experimental intervention study with a quantitative methodology was designed, prospective in nature, and structured as a randomized controlled clinical trial with two parallel groups. This design was chosen to ensure a high level of methodological rigor, as well as the internal and external validity of the results, following the recommendations for conducting clinical trials in health sciences.

The study adopts a multicenter design in which participants, outcome assessors, the principal investigator (PI), and researchers involved in data management and statistical analysis will remain blinded to treatment allocation throughout the study in order to minimize performance, detection, and interpretation biases. Only the physical therapists delivering the interventions are aware of group assignment because of the nature of the treatment administered.

All assessments will be conducted by trained evaluators following standardized measurement procedures. Likewise, the treating physical therapists will receive specific training to ensure consistency in the application of the intervention protocols across the participating centers.

Participant recruitment is expected to be completed by December 2026. Given the 6-month follow-up period, outcome assessments and study follow-up procedures are expected to continue until approximately June 2027, when the final participant completes the last scheduled assessment.

The study is being carried out in three Rehabilitation and Physical Therapy Services located in primary care centers (PCCs) of the Catalan Health Institute (ICS), specifically in Mataró, Sant Andreu (Barcelona), and Drassanes (Barcelona). Conducting a multicenter study in the primary care setting allows the inclusion of a more heterogeneous and representative sample of individuals with LE, thereby increasing the external validity and transferability of the findings to routine clinical practice.

The multicenter design was specifically adopted to enhance recruitment capacity. The participating Rehabilitation and Physical Therapy Services receive referrals from primary care physicians, rehabilitation physicians, orthopedic specialists, and rheumatologists and collectively manage a substantial number of patients with LE each year. Based on historical referral volumes across the participating centers, recruitment of the target sample size within the planned recruitment period is considered feasible.

### 2.2. Recruitment and Blinding

The study population will consist of adults diagnosed with LE attending the Rehabilitation and Physical Therapy Services of three urban primary healthcare centers belonging to the ICS. This setting reflects routine clinical practice, as LE is commonly managed in primary care and rehabilitation services, thereby enhancing the external validity and clinical applicability of the study findings.

Potential participants will be identified through clinical referrals from family medicine, rehabilitation, orthopedics, and rheumatology services. These referrals will be reviewed by the head of each Rehabilitation and Physical Therapy Service to identify potentially eligible individuals.

Subsequently, administrative staff will schedule an in-person appointment with a trained evaluator at each participating center. During this visit, which will last approximately 30 min, eligibility criteria will be verified, baseline clinical data will be collected using the eCAP clinical information system, and all outcome measures will be assessed.

Participants who meet all inclusion criteria and none of the exclusion criteria will be informed about the study by the evaluator using the patient information sheet (PIS). Those willing to participate will provide written informed consent (IC) before any study-related procedure is performed, in accordance with current regulations governing clinical research and data protection.

Participants will be randomly allocated in a 1:1 ratio to one of two parallel groups using a simple randomization procedure. The allocation sequence will be generated by an independent member of the Research Support Unit, who will not be involved in recruitment, outcome assessment, intervention delivery, or data analysis. Allocation concealment will be ensured through sequentially numbered, opaque, sealed envelopes, prepared according to the randomization sequence and opened only by designated administrative staff after participant eligibility has been confirmed and written informed consent has been obtained. The allocation result will be recorded on the treatment form, allowing the treating physiotherapists to identify the assigned study group. Only the physiotherapists responsible for delivering the intervention will have access to treatment allocation information.

Participants, outcome assessors, and researchers involved in the study will remain blinded to group allocation throughout the study. Evaluators will have no access to the randomization sequence or allocation records, and the PI will not participate in treatment delivery or data collection. Researchers involved in statistical analysis will remain blinded to group allocation until the primary analyses are completed.

Participant blinding will be maintained by administering SIS at a non-therapeutic dose in the CG using procedures identical to those of the active intervention. Although several procedures will be implemented to maximize participant blinding, complete blinding cannot be guaranteed, as active SIS treatment may induce visible muscle contractions and sensory responses that could differ from those experienced during sham stimulation.

The intervention group (IG) will receive SIS at a therapeutic dose combined with a structured program of therapeutic exercise and health education. The control group (CG) will receive SIS at a non-therapeutic dose together with the same therapeutic exercise and health education program.

The complete processes of recruitment, allocation, follow-up, and analysis are summarized in Figure 1.

### 2.3. Participants

The reference population will consist of adults aged 18 years or older with LE. The study population will include patients with a clinical diagnosis of LE referred to the Rehabilitation and Physical Therapy Services of the participating centers (Mataró, Sant Andreu, and Drassanes) within the primary care setting of the ICS.

This clinical setting allows the inclusion of a heterogeneous population representative of patients with LE commonly treated in routine clinical practice, thereby enhancing the external validity and applicability of the study findings.

During the baseline assessment visit, a trained evaluator at each participating center will verify eligibility criteria and collect participants’ sociodemographic and clinical characteristics using the eCAP clinical information system and the corresponding assessment instruments. The collected variables will include age, sex, affected limb, dominant hand, symptom duration, employment status, medical history, previous physiotherapy treatments, current analgesic or anti-inflammatory medication use, and other relevant clinical variables that may influence the interpretation of the study results.

### 2.4. Selection Criteria

Eligible participants will be adults aged 18 years or older with a clinical diagnosis of LE established by a qualified healthcare professional and referred to the Rehabilitation and Physical Therapy Services of the participating centers. Participants must have a clinical diagnosis of lateral epicondylalgia established by a qualified healthcare professional and present pain over the lateral epicondyle reproduced during at least one provocative clinical test (Cozen test or chair test) [31].

Exclusion criteria will include medial epicondylalgia, a history of elbow trauma within the previous six months, passive elbow joint range of motion limitations greater than 20 degrees, and corticosteroid or other therapeutic injections in the affected elbow within six weeks prior to study enrollment. Participants with diagnosed systemic conditions that may interfere with study outcomes will also be excluded. These conditions include inflammatory, autoimmune, infectious, or neoplastic diseases, coagulation disorders, and central sensitization syndromes such as fibromyalgia or chronic fatigue syndrome. Participants diagnosed with compressive neuropathies affecting the upper extremity, including radial tunnel syndrome (Frohse arch syndrome), will also be excluded. The diagnosis of these conditions must have been established by a qualified healthcare professional and documented in the participant’s clinical record.

Participants presenting bilateral symptoms may be included; however, only one affected limb will be selected for treatment and outcome assessment to ensure independence of observations.

Regarding intervention safety, individuals with contraindications to SIS treatment, including the presence of pacemakers, implantable defibrillators, or metallic implants in the treatment area, as well as pregnant women, will be excluded.

Finally, individuals with cognitive or sensory impairments that may limit their ability to understand the study procedures, provide IC, or adequately participate in the intervention will not be eligible for inclusion.

### 2.5. Sample Size

Sample size was estimated based on the primary outcome (PRTEE score) analyzed via a linear mixed-effects model (LMM) across four assessment time points. Assuming a standard deviation of 11.3 points [32], and a minimal clinically important difference (MCID) of 7.0 points [33], the standardized effect size was estimated at *d* = 0.62.

Accounting for repeated measures with an intra-subject correlation of 0.50, a sample size of 42 participants per group provides 90% statistical power to detect a significant group-by-time interaction at a two-tailed significance level of α = 0.05.

To account for an anticipated attrition rate of 30% over the 6-month follow-up period across the four measurement time points, a total of 120 participants (60 per group) will be recruited.

This sample size is expected to provide sufficient statistical power to detect clinically meaningful differences in the primary outcome while accounting for the longitudinal design of the study.

### 2.6. Interventions

Participants will be assigned to one of two study groups, IG or CG, according to the previously described randomization procedure. Both groups will receive a multimodal approach based on therapeutic exercise and health education, differing only in the type of application of the SIS.

Health education will be provided individually by the treating physiotherapist during the first treatment session using a standardized educational leaflet. The educational component will include information about lateral epicondylalgia, pain mechanisms, activity modification, ergonomic recommendations, workload management, self-management strategies, prevention of symptom recurrence, and the importance of treatment adherence.

Participants in the IG will receive a treatment consisting of a structured program of therapeutic exercise, health education, and administration of the SIS at a therapeutic dose.

Stimulation intensity is adjusted on a relative scale from 0% to 100% according to patient tolerance and therapeutic objectives.

The intervention will be delivered using the BTL-6000 Super Inductive System Elite (BTL Industries Ltd., Stevenage, UK) device available at the participating centers (Figure 2). The BTL-6000 SIS generates biphasic sinusoidal electromagnetic pulses with a pulse duration of 280 µs and a configurable magnetic field intensity ranging from 0.7 to 2.5 Tesla at the coil surface.

Participants assigned to the IG will receive a 10 min application using the device’s predefined protocol for lateral epicondylalgia. The protocol corresponds to the lateral epicondylalgia program incorporated in the BTL-6000 Super Inductive System Elite device and consists of a sequence of predefined treatment phases with specific time, amplitude modulation, and frequency modulation parameters (Table 1).

Stimulation intensity will be individually adjusted to each participant’s motor threshold, defined as the minimum stimulation intensity required to elicit a muscle contraction. Once the motor threshold has been identified, stimulation intensity may be adjusted within the participant’s tolerance limits to ensure comfort throughout the intervention.

The applicator will be positioned in direct contact with the painful area over the lateral epicondyle and the forearm extensor musculature. Participants will remain seated with the forearm supported, following a standardized positioning protocol. The SIS intervention will be applied after completion of the therapeutic exercise program. The therapeutic purpose of this intervention is to promote pain modulation and improve muscle activation and tissue trophism.

The therapeutic exercise protocol includes upper-limb exercises aimed at improving functionality, strength, and tendon load tolerance. It consists of warm-up exercises, static stretches of the wrist extensor muscles, and progressive strengthening exercises, including isometric and eccentric exercises, as illustrated in Figure 3. The exercises will be prescribed at a frequency of two sets of ten repetitions per exercise. Participants will perform the supervised program twice weekly at the rehabilitation center and will be encouraged to complete the same exercises at home on two additional days per week.

At the first treatment session, the physiotherapist responsible for the intervention will teach and demonstrate all exercises and provide individualized instructions to ensure correct execution. Exercise performance and progression will be monitored and reinforced during each supervised treatment session. Exercise progression will be individualized according to participant tolerance, symptom response, and exercise performance. Progression through the three predefined exercise levels will be supervised weekly by the treating physiotherapist. Exercise load, resistance, movement amplitude, and task complexity may be increased provided that pain during exercise does not exceed 5 points on a 10-point VAS and no symptom exacerbation is reported within 24 h after exercise performance. Resistance will be provided using elastic bands (Thera-Band^®^, 1.3–2.1 kg resistance levels), tennis balls, free weights, and household objects such as water bottles, depending on the exercise and progression level.

Adherence to the exercise program will be monitored using exercise log sheets completed throughout the intervention period. Participants will record both supervised and home-based exercise sessions. At the end of the intervention, adherence will be quantified as the total number of exercise sessions completed relative to the prescribed program.

The protocol will conclude with a brief self-massage of the affected forearm extensor musculature. Participants will be instructed by the treating physiotherapist on the appropriate self-massage technique, which consists of gentle longitudinal and circular movements over the affected muscle groups with the aim of promoting muscle relaxation and increasing local tissue comfort. A brief self-massage will be included to promote self-management, improve local comfort, and facilitate adherence to the home-based component of the intervention. The self-massage procedure will be standardized and applied equally in both study groups.

Participants assigned to the CG will receive the same therapeutic exercise and health education protocol, together with SIS delivered at a non-therapeutic dose according to the manufacturer’s recommendations. The application will last 10 min and will be delivered using a frequency of 1 Hz, a pulse/pause ratio of 1 s/59 s, and a fixed intensity of 5%. The same device, treatment duration, participant positioning, and therapeutic environment used in the IG will be maintained. Participants will not be informed which stimulation parameters correspond to the active treatment. This configuration was selected to maintain participant blinding while minimizing the probability of meaningful physiological or clinical effects. However, the applicator will be positioned approximately one handbreadth from the skin, thereby avoiding effective tissue stimulation.

To further support participant blinding, SIS applications will be administered individually in a dedicated treatment room. Participants will not have access to treatment sessions of other participants and will not be able to observe the stimulation procedures applied to other study participants.

The intervention will last a total of four weeks, with two sessions per week, for a total of eight sessions per participant. The sessions will be conducted in the Rehabilitation and Physical Therapy Services of the participating centers by physical therapists with clinical experience who have received specific training in the study protocol to ensure uniform intervention delivery across centers. Both the IG and the CG will receive the same number and duration of treatment sessions, as well as the same therapeutic exercise and health education program. The only difference between groups will be the dose of SIS administered.

To ensure methodological quality and the study’s replicability, a standardized protocol will be used for all participants, and the parameters of the intervention’s application, as well as treatment adherence and the number of sessions performed, will be recorded.

To enhance intervention reproducibility, detailed stimulation parameters of the predefined lateral epicondylalgia SIS program are presented in Table 1.

The protocol corresponds to the predefined lateral epicondylalgia program incorporated in the BTL-6000 Super Inductive System Elite device. Each section represents a predefined phase of the treatment sequence with its corresponding duration and amplitude/frequency modulation parameters. Stimulation intensity will be individually adjusted to each participant’s motor threshold, defined as the minimum stimulation intensity required to elicit a visible muscle contraction.

Figure 2 shows the BTL-6000 Super Inductive System Elite used in the three centers assigned to the study.

Figure 3 illustrates the therapeutic exercise protocol designed for the study participants diagnosed with LE.

### 2.7. Outcome Measures

Outcome assessments will be performed at four time points: baseline (T0), immediately after completion of the intervention (T1), and at 3-month (T2) and 6-month (T3) follow-ups. All assessments will be conducted by trained evaluators blinded to group allocation and following standardized measurement procedures. The outcome variables and assessment instruments are summarized in Table 2.

The primary outcome will be pain and functional disability associated with LE, assessed using the Patient-Rated Tennis Elbow Evaluation (PRTEE), a condition-specific and validated instrument for patients with LE [33]. A reduction of approximately 7 points on the PRTEE score will be considered clinically meaningful based on previously reported minimal clinically important difference (MCID) values. The PRTEE total score will be considered the primary outcome measure of the study.

Secondary outcomes will include pain intensity, pressure pain sensitivity, elbow mobility, grip strength, upper-limb function, and health-related quality of life. Pain intensity will be measured using the VAS, a reliable and widely used instrument for pain assessment [34]. A reduction of 1.5–2.0 points or approximately 30% from baseline will be considered clinically meaningful. Pressure pain sensitivity will be assessed using pressure algometry, a method with demonstrated inter-rater reliability for measuring pressure pain thresholds [9].

Elbow range of motion in flexion, extension, pronation, and supination will be measured using standard goniometry, a valid and reliable method for assessing joint mobility [35,36]. Handgrip strength will be evaluated using a JAMAR^®^ dynamometer, considered the reference standard for grip strength assessment [37,38]. Changes of approximately 5–6 kg or 15–20% relative to baseline will be considered clinically relevant. The mean of three consecutive attempts will be recorded following standardized testing procedures.

Upper-limb function will be assessed using the Quick Disabilities of the Arm, Shoulder and Hand questionnaire (Quick DASH), which has demonstrated satisfactory validity and reliability for evaluating upper-extremity disability [39]. A change of 14–16 points will be considered clinically meaningful. Health-related quality of life will be assessed using the EQ-5D-5L questionnaire [40,41,42,43]. Changes of approximately 0.07–0.10 points in the EQ-5D-5L index score will be considered clinically relevant.

Additional variables related to the therapeutic process and overall clinical impact will also be recorded. Treatment adherence will be calculated using attendance records and exercise log sheets completed throughout the intervention period.

Adherence to the home exercise program will be monitored using a patient-completed exercise log. Temporary work disability will be collected from the electronic clinical record and will include both the number of episodes and their duration in days. Information regarding analgesic and anti-inflammatory medication use will be obtained during each assessment visit and verified through the electronic clinical record whenever possible. Any adverse events occurring during the intervention or follow-up period will be systematically recorded by the treating physiotherapists and documented in the study database.

The effectiveness of participant blinding will be assessed immediately after completion of the intervention. Participants will be asked to indicate whether they believe they received the active intervention, the control intervention, or if they are uncertain. In addition to reporting the proportion of correct responses, the Bang Blinding Index will be calculated to quantify the success of participant masking. Blinding will be considered successful if participants are unable to correctly identify their treatment allocation at a rate greater than that expected by chance.

All outcome measures will be assessed by trained physical therapists with clinical experience and specific training in the administration of the assessment instruments used in this study. Standardized measurement procedures will be followed to ensure consistency, reliability, and methodological rigor throughout the evaluation process.

For interpretation of clinical relevance, a reduction of approximately 7 points in the PRTEE score, 1.5–2.0 points (or approximately 30%) in the VAS score, 5–6 kg (or 15–20%) in grip strength, 14–16 points in the QuickDASH score, and 0.07–0.10 points in the EQ-5D-5L index score will be considered clinically meaningful based on previously reported minimal clinically important difference (MCID) values [44,45,46].

### 2.8. Safety Monitoring and Adverse Events

The safety of participants will be monitored throughout the intervention and follow-up periods. All adverse events potentially related to SIS, a noninvasive and generally well-tolerated therapy, will be systematically recorded. The treating physical therapists will be responsible for detecting, documenting, and reporting each adverse event, including its characteristics, duration, severity, and potential relationship to the intervention.

Any unexpected clinical sign or symptom, or any worsening of pre-existing symptoms occurring during the study period, will be considered an adverse event. Particular attention will be paid to known adverse effects associated with SIS treatment, including temporary muscle soreness, transient muscle spasms, temporary tendon or joint pain, and local erythema. If necessary, treatment discontinuation will be considered to ensure participant safety and appropriate clinical management.

No specific pharmacovigilance system is planned because medication is not part of the intervention protocol. However, participants’ medication use will be recorded throughout the study and considered during data analysis.

### 2.9. Statistical Analysis

Statistical analyses will be conducted using SPSS v.26.0 software. A significance level of *p* < 0.05 will be considered, corresponding to a 95% confidence interval. Data will be collected and managed using REDCap (Research Electronic Data Capture) electronic data capture tools [47,48].

A descriptive statistical analysis of continuous variables will be performed using means and standard deviations or median and interquartile range, depending on data distribution. Categorical variables will be expressed as absolute frequencies and percentages. Data normality will be assessed using the Shapiro–Wilk test and graphical methods.

Baseline comparability between groups will be analyzed using Student’s *t*-test for independent samples or the Mann–Whitney *U* test for continuous variables, depending on data distribution, and the chi-square test for categorical variables.

The primary outcome (PRTEE score) will be analyzed using a linear mixed-effects model for longitudinal data. Treatment group, time, the group-by-time interaction, and study center will be included as fixed effects, while participant will be included as a random effect to account for the correlation between repeated measurements within the same participant. The group-by-time interaction will be the primary effect of interest and will be used to determine whether changes differ between the intervention and control groups. The 6-month follow-up assessment (T3) will be considered the principal time point for evaluating treatment durability.

An intention-to-treat (ITT) analysis will be performed, including all randomized participants in the groups to which they were originally allocated, regardless of treatment adherence or study completion. In addition, a per-protocol analysis will be performed as a sensitivity analysis. Participants who attend at least 80% of the scheduled treatment sessions and do not present major protocol deviations will be included in the per-protocol population. The extent and pattern of missing data will be described by treatment group and assessment time point. Baseline characteristics of participants with and without missing outcome data will be compared descriptively to explore potential patterns of missingness and inform the assumptions underlying the missing-data approach. Missing data will be handled using multiple imputation under a missing-at-random assumption. A complete-case sensitivity analysis will be performed by excluding participants with missing data to assess the robustness of the results of the primary analysis. All secondary outcomes will be analyzed using similar models but will be considered exploratory. The results from secondary outcome analyses will therefore be interpreted as supportive evidence for the primary findings.

### 2.10. Ethical Considerations

The study will be conducted in accordance with the ethical principles set forth in the Declaration of Helsinki (latest version) and the guidelines for good clinical practice (CPMP/ICH/135/95), as well as with the applicable legislation on biomedical research and personal data protection.

The study protocol has been reviewed and approved by the Clinical Research Ethics Committee of IDIAP Jordi Gol (CEIm IDIAP Jordi Gol; approval number 25/125-ACps) and has been prospectively registered at ClinicalTrials.gov (NCT07495254).

All participants will be informed in a clear and detailed manner about the study’s objectives, procedures, benefits, and potential risks using the patient information sheet. Participation will be completely voluntary, and participants must sign the informed consent form before being enrolled. The right to withdraw from the study at any time will be guaranteed without any impact on their routine healthcare.

The participants’ personal data will be processed confidentially in accordance with the General Data Protection Regulation (EU) 2016/679 and Organic Law 3/2018 on the Protection of Personal Data and Guarantee of Digital Rights. All data will be coded and anonymized to ensure participant confidentiality, and will only be accessible to the authorized research team.

The collected information will be stored in secure systems of the ICS and retained for the period established by applicable regulations. No international data transfers or disclosures to third parties are planned, except in cases provided for by law.

## 3. Conclusions

This study protocol describes a randomized, multicenter clinical trial in which participants, outcome assessors, the principal investigator, and researchers involved in data management and statistical analysis are blinded.

It is designed to evaluate the effectiveness of the SIS combined with therapeutic exercise and health education in people with LE.

The main objective is to determine the added value of SIS within a multimodal treatment approach in which therapeutic exercise represents the core intervention.

The proposed methodological design, including random allocation, placebo-controlled intervention, blinding procedures, validated outcome measures, and short- and medium-term follow-up assessments, is intended to provide robust evidence regarding the potential effectiveness and durability of treatment effects.

By evaluating clinically relevant outcomes such as pain, functional disability, grip strength, mobility, and health-related quality of life, this study seeks to reduce current uncertainty regarding the role of SIS in the management of LE and to support the development of more standardized, evidence-based physiotherapy protocols, particularly in primary care settings.

Although health-related quality of life and work absenteeism data will be collected, a formal cost-effectiveness analysis is beyond the scope of the present study. Nevertheless, these variables may provide valuable information for future research exploring the economic implications and potential cost-effectiveness of SIS-based interventions in individuals with lateral epicondylalgia.

## Figures and Tables

**Figure 1:**
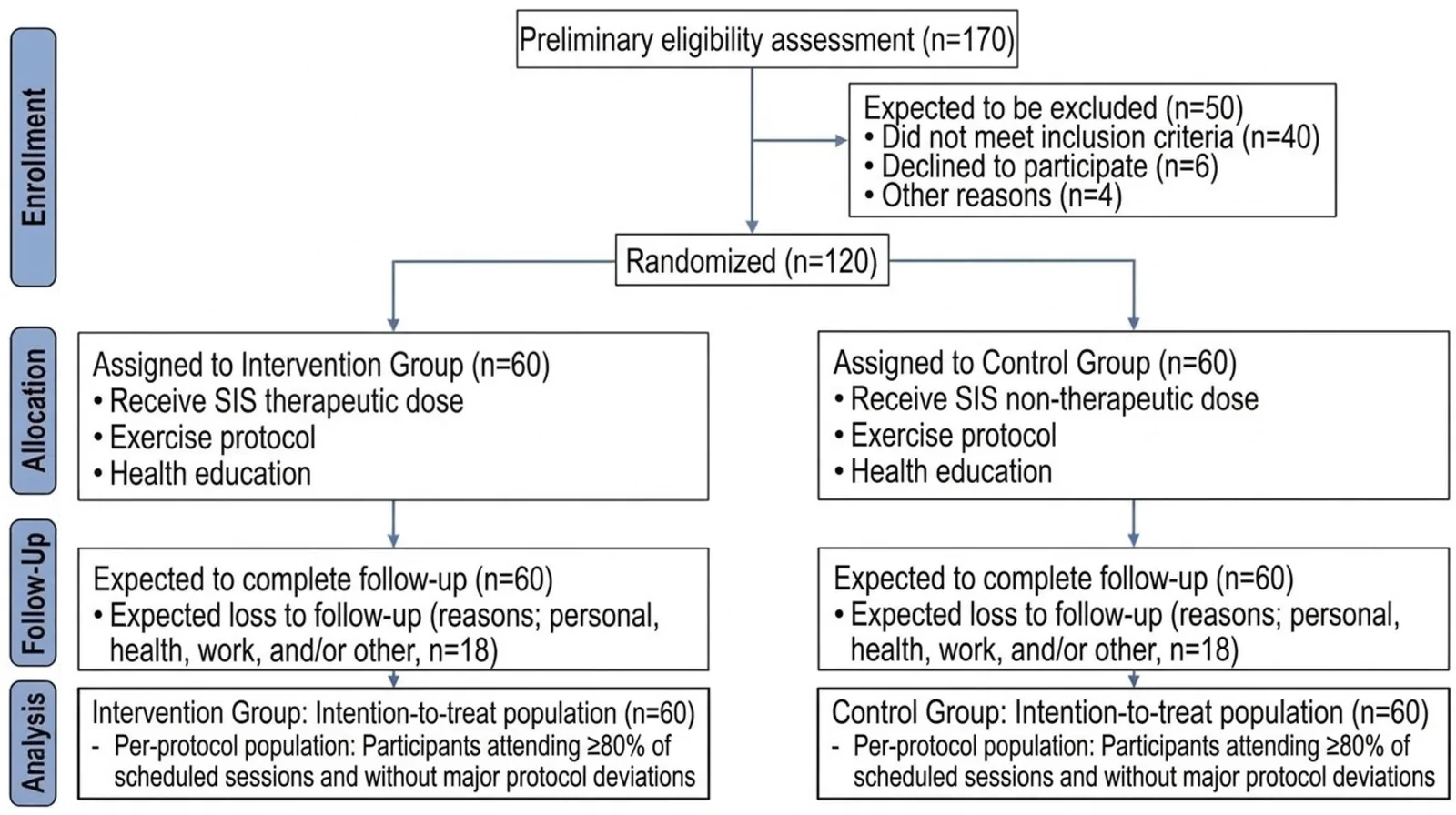
Planned CONSORT flow diagram: This diagram represents the planned participant flow; all “planned n” values will be replaced with actual numbers upon trial completion.

**Figure 2:**
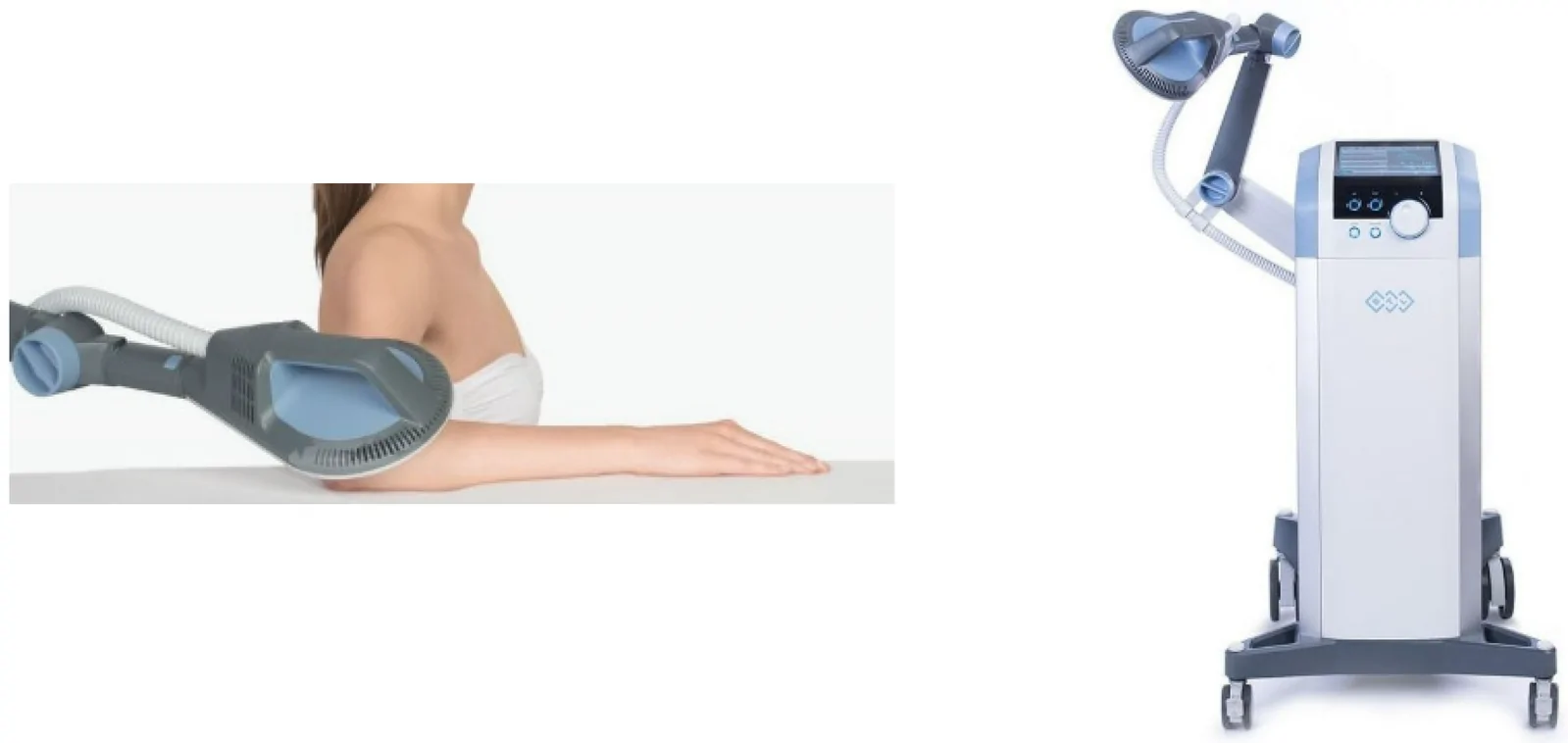
BTL-6000 Super Inductive System Elite.

**Figure 3:**
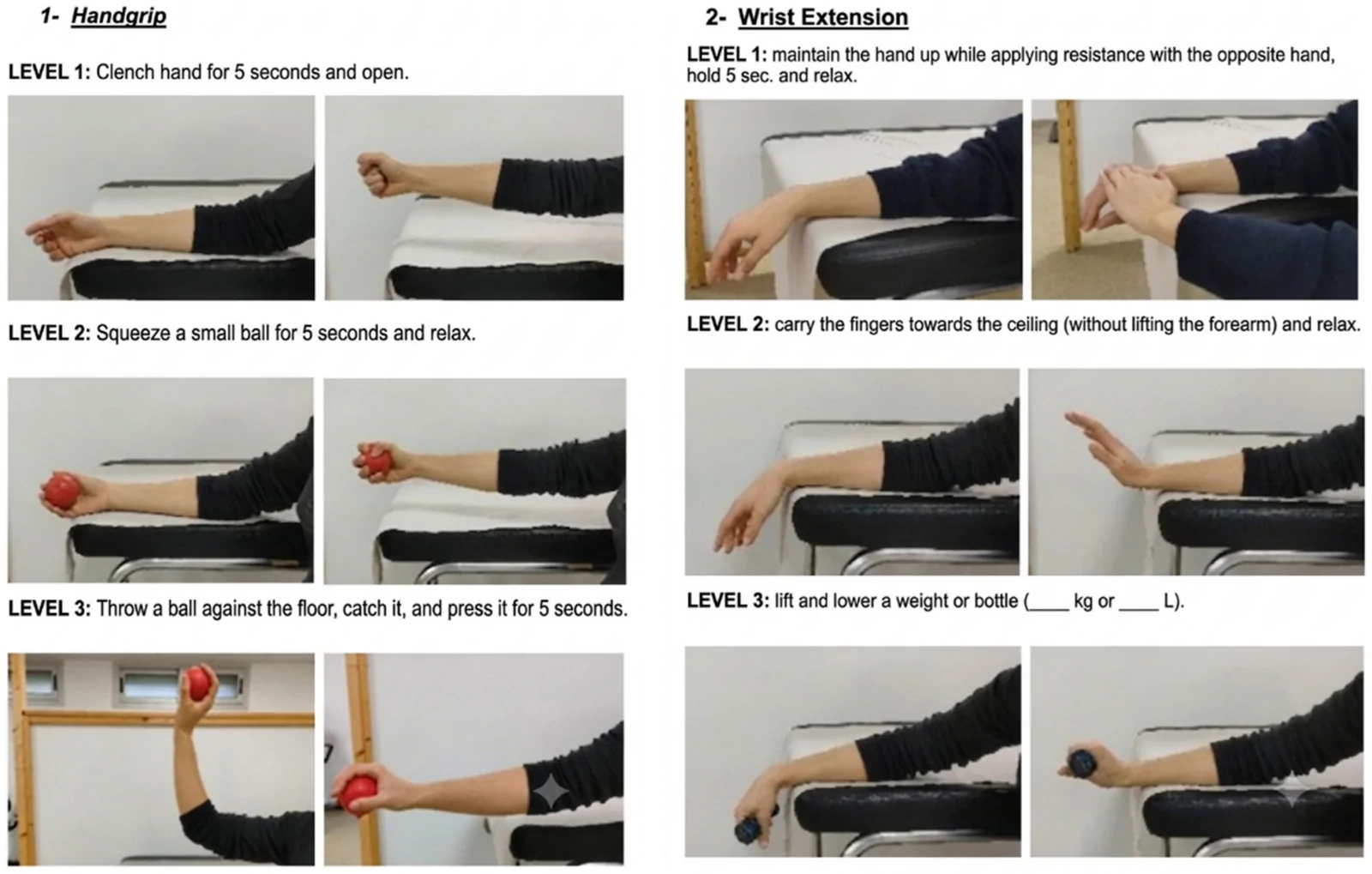

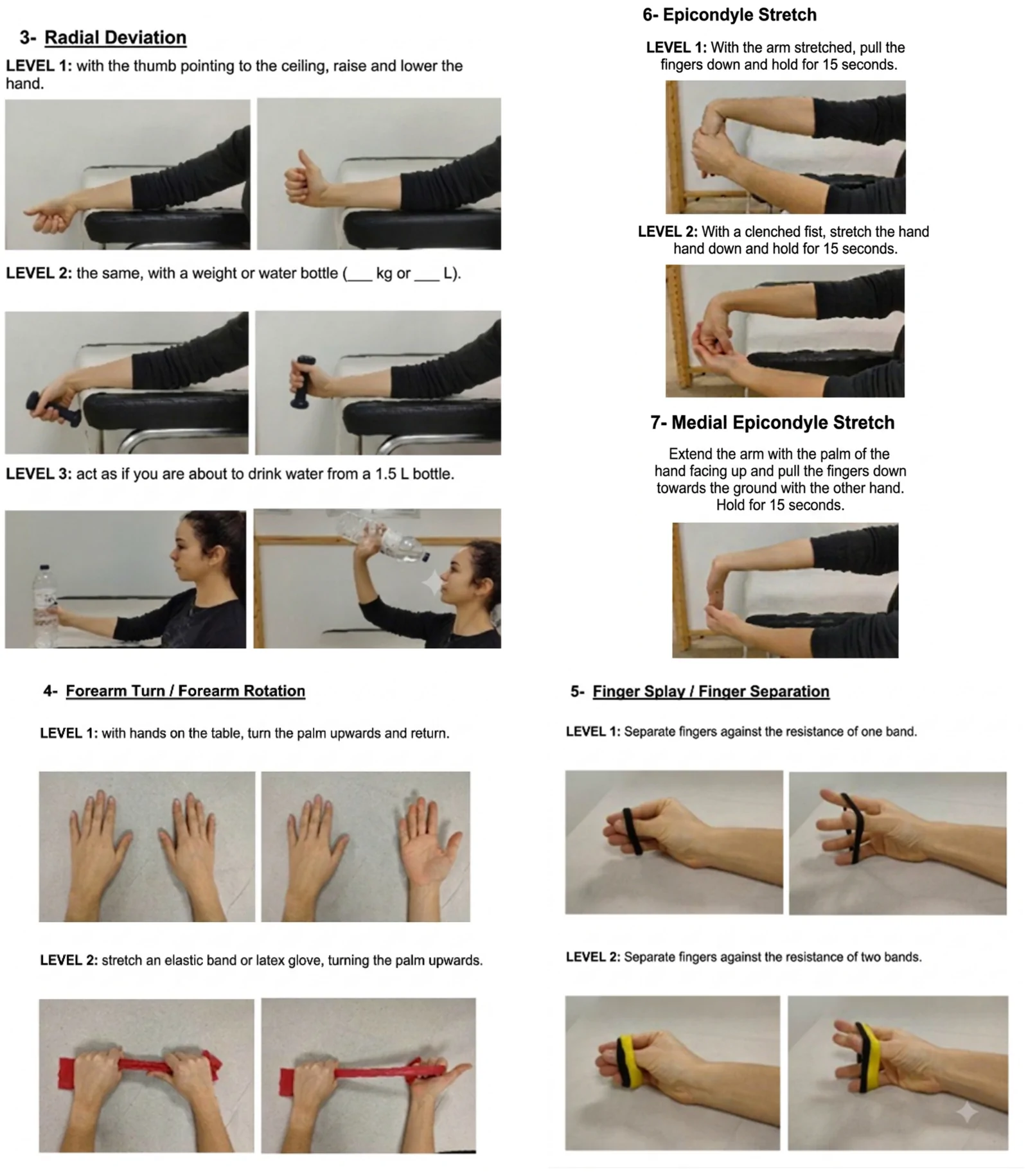
Therapeutic exercise program for lateral epicondylalgia.

**Table 1:** Therapeutic SIS application parameters for participants in the IG.

Section	Section Time	Amplitude Modulation	Frequency Modulation
Section 1	30 s	Pulse = 1 s Pause = 1 s AMT * = Rectangular	Frequency 1 = 5 Hz (2 s) Frequency 2 = 50 Hz (2 s) FMT ** = Rectangular
Section 2	30 s	Pulse = 1 s Pause = 0 s AMT = Rectangular	Frequency = 1 Hz FMT = None
Section 3	3 min	Pulse = 12 s Pause = 6 s AMT = Rectangular	Frequency 1 = 5 Hz Frequency 2 = 10 Hz Period *** = 12 s FMT = Sinusoidal
Section 4	1 min	Pulse = 1 s Pause = 1 s AMT = Rectangular	Frequency = 50 Hz FMT = None
Section 5	3 min	Pulse = 12 s Pause = 6 s AMT = Rectangular	Frequency 1 = 5 Hz Frequency 2 = 10 Hz Period *** = 12 s FMT = Sinusoidal
Section 6	1 min	Pulse = 1 s Pause = 1 s AMT = Rectangular	Frequency = 50 Hz FMT = None
Section 7	1 min	Pulse = 1 s Pause = 0 s AMT = Rectangular	Frequency = 1 Hz FMT = None

Abbreviations: AMT *, amplitude modulation type, refers to the waveform’s shape in terms of pulse and pause times. The device can work with rectangular, trapezoidal, sinusoidal, or staircase waves. FMT **, frequency modulation type, refers to the waveform’s shape in terms of time at each frequency. The device can work with no frequency (“none,” meaning the frequency is constant), as well as with rectangular, trapezoidal, sinusoidal, or random waves. Period ***, the period used with sinusoidal FMT, refers to the time that elapses between Frequency 1 and Frequency 2, sweeping through all intermediate frequencies.

**Table 2:** Outcome measures and evaluation tools.

Assessment Tools	Variables
Primary Outcome	
PRTEE	Pain and functional disability
Key Secondary Outcomes	
VAS	Pain intensity
Pressure Algometry (PAIN TEST^TM^ FPX 50)	Pressure pain threshold
Standard Goniometer	Elbow range of motion
JAMAR^®^ Hydraulic Dynamometer	Grip strength
Quick DASH Questionnaire	Upper-limb function
EQ-5D-5L	Health-related quality of life
Additional Outcomes	
Session Log/Self-Report	Treatment adherence
Clinical Records	Work absenteeism (number and duration)
Patient Report	Medication use
Adverse Event Log	Adverse events
Masking Questionnaire	Masking effectiveness

Abbreviations: PRTEE, Patient-Rated Tennis Elbow Evaluation; VAS, Visual Analog Scale; Quick DASH, Quick Disabilities of the Arm, Shoulder and Hand; EQ-5D-5L, EuroQol 5-Dimension 5-Level questionnaire. Outcome measures will be collected at baseline (T0), immediately after the intervention (T1), and at 3-month (T2) and 6-month (T3) follow-ups by trained evaluators blinded to treatment allocation. PAIN TEST^TM^ FPX 50, digital pressure algometer; JAMAR^®^, hydraulic hand dynamometer. Health-related quality of life will be assessed using the EQ-5D-5L questionnaire.

## Data Availability

Data generated during the study will be available from the corresponding author upon reasonable request following publication of the study results, in accordance with applicable ethical and data protection regulations.

## Abbreviations

The following abbreviations are used in this manuscript:

ADL	Activities of Daily Living
CG	Control Group
eCAP	Primary Care Coordination and Management System
EQ-5D-5L	EuroQol 5-Dimension 5-Level
IC	Informed Consent
ICS	Institut Català de la Salut—Catalan Institute of Health
IG	Intervention Group
LE	Lateral Epicondylalgia
ITT	Intention-To-Treat
MCID	Minimal Clinically Important Difference
NRS	Numeric Rating Scale
PCC	Primary Care Center
PRTEE	Patient-Rated Tennis Elbow Evaluation
PI	Principal Investigator
PIS	Patient Information Sheet
QuickDash	Quick Disabilities of the Arm, Shoulder and Hand
REDCap	Research Electronic Data Capture
SIS	Super Inductive System
SPSS	Statistical Package for the Social Sciences
TENS	Transcutaneous Electrical Nerve Stimulation
VAS	Visual Analog Scale
